# Regenerative topical skincare: stem cells and exosomes

**DOI:** 10.3389/fmed.2024.1443963

**Published:** 2024-10-15

**Authors:** Amy Forman Taub

**Affiliations:** ^1^Institute Advanced Dermatology, A Forefront Dermatology Practice, Lincolnshire, IL, United States; ^2^Department of Dermatology, Institute Northwestern University Medical School, Chicago, IL, United States

**Keywords:** skin, aesthetic, regenerative, stem cells, exosomes

## Abstract

Regenerative medicine and its offshoot, regenerative aesthetics, have been hot topics over the past 15 years. Studies with heterochronic parabiosis and others pointed to a circulating factor that could rejuvenate aging tissues. Stem cells are known to have regenerative powers, but they are difficult to extract, grow in culture or maintain. Exosomes (EVs), extracellular vesicles from 30 to 150 nm, have been discovered to be a primary form of communication between tissues. Using stem cell supernatants to generate desirable EVs has become a heralded treatment for aesthetic treatments. Preclinical studies with EVs show many benefits including improving the function of fibroblasts and healing wounds more rapidly. Clinical studies with EVs in aesthetics are very few. Thus, the excitement generated by EVs should be tempered with realism about the lack of available treatment products as well as the lack of scientific proof.

## Introduction

As humans, almost every cell in our body has been replaced countless times over the course of our lives, with a few key exceptions such as brain neuronal cells (1). The complex process of aging results in the gradual decline of the body’s metabolism and its ability to renew, repair, and regenerate, leading to compromised function which further hampers this capability, until the body is no longer able to sustain life.

The focus of the rapidly evolving field of regenerative medicine is, “to restore the functionality of tissues, organs, or body parts damaged by trauma, disease or aging.” (2) Regenerative aesthetics is an offshoot of regenerative medicine, harnessing that emerging fields’ concepts to aesthetic ends. These fields are intertwined; advances in one have often led to advances in the other. Much of the rapidly proliferating armamentarium is the same (3).

Our nascent understanding that something in the circulation is critical for reversal of aging was ignited by renewed experimentation over the past 15 years with heterochronic parabiosis, i.e., surgically attaching a young and an old mouse (4). One experiment showed that even cognitive ability of an old mouse can be improved by sharing young mouses plasma- the old mouse gained spatial orientation of a young mouse after they were conjoined (5).

The search for identification of the component(s) in the circulation responsible for the youthifying of the older tissues has been active and has focused on stem cells, peptides, growth factors, mRNA, miRNA and other soluble biologically active molecules. There is palpable excitement about the newest component to be identified, exosomes, designated as EVs, that are being categorized and tested in pre-clinical studies as well as early clinical studies.

Twenty years ago, revolutionary skin care was introduced that contained the supernatant of a scaffold of cultured neonatal human fibroblasts, ushering in the “biologics” era in topical aesthetics (6). Over 110 active growth factors were identified, and the resulting skin care product was heralded as a breakthrough and is still in use today. Many tried to find the one or few growth factors that were the most important, but others argued it was the “physiologic” preparation that was responsible for the effects (7). But recently, it was found that this same product contained EVs, and that some of the effects seen with the product, which had been ascribed to growth factors, may have been due to EVs (8).

This is a mini review: the purpose is to capture a snapshot of current literature as it pertains to EVs for aesthetic purposes. There is an extensive basic science literature regarding wound healing with EVs; there is overlap between wound healing and aesthetic concerns such as stimulation of collagen production. The review below of the latter is representative only. There are less than 20 clinical trials focusing on skin being performed, and a handful are published on aesthetic concerns, all of which are discussed below.

## Stem cells

Multipotent stem cells were discovered by Till and McCulloch in 1961 (9). The adult stem cell plays a key role in the maintenance and restoration of tissue and organs. Stem cells regulate all 4 phases of healing and repair: hemostasis, inflammation, proliferation and remodeling (10). But stem cells accumulate DNA damage with aging resulting in impaired protein activity, cell function and normal organ physiology. A hallmark of aging is exhaustion of the endogenous stem cell population, resulting in a lessened ability to repair.

Also, stem cells are not abundant and are difficult grow in culture^1^. They require a hypoxic environment to stave off differentiation (11). They must be extracted from tissues and kept frozen. Thus, in addition to the regulatory obstacles involved in their use, populations of stem cells are difficult and costly to obtain and maintain. No skincare product actually contains stem cells (12).

This shifted the narrative from extraction of stem cells with implantation into aging tissues to utilizing growth factors, peptides and EVs, all of which are non-living entities that can be more easily stored and reused. In addition, they exhibit lower immunogenicity and appear to be the actual effectors of downstream molecular pathways, reducing inflammation and simulating youth-associated biological processes.

Within the skin, basal stem cells produce keratinocytes, plus 10 types of hair follicle stem cells. Among these, LGR 6+ stem cells are key, serving as the primary source from which all skin cells are produced during development and shortly after birth. These are preserved in the isthmus zone of hair follicles where they remain dormant until activated during wound healing (13). Another method for anti-aging in the skin is to stimulate our own endogenous stem cells in the epidermis with a messenger cytokine called a defensin. These are deployed by neutrophils after wounding to turn on the LGR-6+ stem cells that lay dormant in our follicles, stimulating them to produce a new epidermis. In a randomized controlled trial of 30 people, a product containing defensins 3 and 5 caused a statistically significant improvement in wrinkles and pores vs. control substance on facial skin, as well as epidermal thickness as measured by biopsy (14). This is a unique and potentially valuable way of stimulating our own endogenous cells to become more active and produce new epidermis. It is also unique in that it is stimulating stem cells that have been sequestered in the hair follicle for use during wounding, so they may be subject to less senescent influence than other stem cells in everyday usage.

Stem cells are still valued greatly due to their highly regenerative nature. However, instead of using the stem cell themselves to be infused or used topically on the skin, the supernatant from cultured stem cells are often used to extract the downstream effectors they produce: EVs, growth factors, proteins, cytokines, mRNA, and miRNA.

## Exosomes (EVs)

Typically, about 1/200th the size of the average cell, EVs are extracellular vesicles that occur through exocytosis from cells. They contain growth factors, cytokines, mRNA, miRNA and other cell signaling molecules. According to a 2018 position statement updating the guidelines of the International Society for Extracellular Vesicles (15), EVs are “Ubiquitous nanoparticles naturally released from cells, delimited by a lipid bilayer and that cannot replicate. Thought to be extracellular garbage at one time and now considered to be our information highway between tissues.” Since EVs are used for cell-to-cell communication, it could be postulated that their contents, containing multiple different but physiological building blocks, are instructions to and material for the recipient cell, somewhat akin to a recipe.

EVs are typically isolated by collecting the supernatant from the cell culture growth medium, followed by sequential centrifugation at increasing forces. The medium is filtered, and the resulting pellet is resuspended. To confirm the resultant product contains EVs, transmission electron microscopy and western blotting are typically employed to assess morphology and protein content that have been characterized and accepted in the scientific community as EVs (16).

Although EVs may be extracted from any cell population, those derived from stem cells appear to be the most potent. The best source of EVs for skin rejuvenation is hotly debated. Mesenchymal stem cell associated EVs, derived from “Age 0” or perinatal stem cells are particularly prized since they are believed to have special regenerative powers as well as being less immunogenic (17). These MSCs may be derived from the acellular Wharton’s Jelly in human umbilical cords, placental tissue or human skin fibroblasts from neonates. The latter are proposed to be more specific for skin regeneration as they are a component of the skin itself (8). Platelets are another source of EVs for topical rejuvenation, based on their role in initiating wound healing (18). One group has developed a proprietary, commercially available, method of extracting platelet-derived EVs that have optimal regenerative properties (19). Other important sources are adipose derived stem cells (20).

Stem cells and EVs are highly regulated by the US Food and Drug Administration (FDA), and both require FDA approval for legal use. Currently, the only stem cell products that are FDA-approved for use in the United States consist of blood-forming stem cells (also known as hematopoietic progenitor cells) that are derived from umbilical cord blood. These products are approved for use in patients with disorders that affect the production of blood (i.e., the “hematopoietic” system) but they are not approved for other uses. There are currently no FDA approved exosome products (21). However, there is a growing body of evidence of their efficacy.

## Pre-clinical studies on EVs in skin

Many studies in skin with EVs focus on wound healing. Some also utilize EVs as a carrier for molecules that have been known to accentuate healing (Table 1). Hydrogels are thought to be an effective vehicle for producing wound healing when paired with EVs (22, 23).

**Table 1:** Representative sampling of preclinical studies on Evs in skin.

Reference #	Test product	End points	Subject
Preclinical			
Liang et al. (24)	ADSCM derived EVs	Improve human fibroblasts migration, increase production of collagen type I, inhibit expression of 147 p53, p21 and p15	Human fibroblasts
Nguyen et al. (25)	ADSCM derived EVs − +niacinamide and resveratrol	Decreased ROS (more than Evs or AOX alone)	*In vivo* fibroblasts
Zhu et al. (26)	MSC derived EVs	Improved fibrosis, macrophage activity, epithelial regeneration and collagen deposition	Rodents
Zhu et al. (26)	Human Whartons Jelly (MSC) Derived EVs	Reduction MMPs, galactosidase and Ros, Increased cell viability, Collagen 1 and 3	Senescent FB
Kim et al. (27)	MSCCM derived EVs	Increased Collagen 1 and 3, Decreased MMP	Human skin

Adipocyte derived MSC EVs were shown to improve human fibroblasts migration, increase production of collagen type I, and reduce b-galactoside, ROS, as well as inhibit expression of p53, p21 and p15, proteins associated with inflammation and senescence (24). In another study, adipose derived stem cells EVs were paired with niacinamide and resveratrol and used *in vivo* to study effects on human fibroblasts. The fibroblasts incubated with the loaded EVs were able to reduce reactive oxygen species by 38%, higher than the EVs (13%) or antioxidants alone (19%), indicating some synergy (25).

Preclinical work in mice and rats have shown that EVs derived from mesenchymal stem cells can result in reduced fibrotic wound healing, increased collagen synthesis, improved macrophage function, induce fibroblastic migration and activation, enhance epithelial regeneration and optimize collagen deposition (26).

*In vivo* study of mesenchymal EVs from human-derived Whartons jelly used on a population of senescent fibroblasts (FB) showed that there was a significant difference over control FB in reduction of MMPs and galactosidose and ROS as well as improvement of cell viability, expression of collagen 1 and 3 and time to heal scratches. These beneficial effects were amplified by adding hydrolyzed collagen oligopeptides (HCOPs) (26).

When human skin was exposed to MSCCM-derived EVs, they were not only incorporated into the skin but produced statistically significant increases (over control skin) in Collagen 1 and 3, and elastin as well as reduction in MMPs. This was also significantly more than their incubation with the MSCCM themselves (27).

This is not an exhaustive list but a sampling that is representative of EVs research in skin.

## Clinical studies on EVs in skin)

Clinical studies for topical products utilizing EVs are few (Table 2).

**Table 2:** Clinical studies on EVs.

Reference #	Test product	End points	Subject
Clinical			
Proffer et al. (19)	Human platelet Derived Exosomes (HPE)	Reduced wrinkles, redness and melanin	Human facial skin
Nguyen et al. (25)	ADSCE	Reduced pore size, wrinkling and TEWL	Human hands
Park et al. (28)	ADSCE after microneedling X 3 post treatment on treatment day only	Increased GAIS, wrinkles, elasticity and hydration, decreased melanin, over side of face not treated with EVs	Human facial skin
Kwon et al. (29)	ADSCE after plus 2 days on CO2 ablated skin	32.5% scar improvement vs. 19.5% for no treatment after; reduced healing time on treated side	Human acne-scarred facial skin

A prospective, single-arm, non-randomized, longitudinal trial out of the Mayo Clinic in 2022 studied healthy subjects (*n* = 56, age range 40–85 years, Fitzpatrick skin types I-IV) with mild to moderate global wrinkles and fine lines (19). Each performed a six-week regimen of products containing human platelet extract serum (HPE), evaluated via specialized 3D photography and imaging, as well as participant questionnaire. A unique method was used to purify the EVs which minimized exosome damage during the process. The product was lyophilized and exhibited good shelf life. Statistically significant outcomes were noted including reduced wrinkles, redness, and melanin, as well as improved luminosity and more even skin coloration. The product was shown to be safe, effective, well-tolerated and was well-liked by study subjects.

A study of adipocyte stem cell derived EVs on the human hands of 3 individuals showed a reduction in pore size and improvement in texture and transepidermal water loss (TEWL) after 8 weeks of use (25).

A split-face RCT was performed on 28 subjects who underwent facial microneedling every 3 weeks for 3 treatments, followed by either application of Adipose derived stem cell EVs (AdSCe) or control. There was a statistically significant increase in the GAIS as well as wrinkles, elasticity, hydration and decreased melanin as measured by PRIMOS, cutometer and mark-vu on the ADSCE treated side (28). In one other split-face RCT of subjects with acne scarring, all were treated 3 times with CO2 fractional laser and followed for 12 weeks. One side was treated with ADSCE post procedure and for 2 more days, whereas the other side was treated with control gel. There was an overall 32.5% improvement on the ADSCE treated side whereas the control group was only 19.5%. Also, all scar types had significant improvement, moreover, there was a shorter downtime of the ASDC treated side (29).

## Discussion

Currently, 217 clinical trials related to EVs are listed on ClinicalTrials.gov, with 14 focusing on cutaneous concerns such as wound healing, atopic dermatitis, and melanoma, and only a few addressing aesthetic issues like laser healing, hair loss, and melasma. Despite frequent discussions about EVs in skincare and post-procedure contexts at dermatology conferences, meaningful data from diverse trials is needed for EVs to be fully integrated into our aesthetic practices (30). There is no consensus on purity of preparations, optimal source(s), nor dosage or yield, let alone safety in humans. Although there are exciting possibilities based on preclinical studies and a few clinical studies, we are far from rigorous scientific studies that will reassure the practitioner as well as the FDA that preparations with EVs are safe and effective for short or long term usage.

## Footnotes

^1^ Available at: https://www.unmc.edu/stemcells/stemcells (Accessed October 7, 2024)
